# Coping and Co-creation: One Attempt and One Route to Well-being. Part 2. Application to Identity and Social Well-being

**DOI:** 10.11621/pir.2021.0314

**Published:** 2021-09-30

**Authors:** Florence C. Denham, Tjeerd C. Andringa

**Affiliations:** a University of Leiden, Leiden, Netherlands; b University of Groningen, Groningen, Netherlands

**Keywords:** Well-being, ontological security, psychological safety, coping, co-creating, core cognition, identity, authoritarianism

## Abstract

**Background:**

This is the application part of a two-part paper that starts from the assumption that core cognition for promoting agent well-being is shared by all living beings. In Part 1, we derived a number of key terms of core cognition and two behavioral ontologies: coping and co-creation.

**Objective:**

Our first aim is to extend the conceptual framework and two behavioral ontologies, while explaining, from first principles, the observed basic structure in identity development. The second is to apply core cognition on a metatheoretical level to explain how the two theories about fostering well-being show the characteristic features of our two behavioral ontologies.

**Results:**

We demonstrate that the four different combinations of coping, co-creation, adequacy, and inadequacy explain from first principles the underlying structure of identity. Among other things, these accurately leads us to the defining features of authoritarianism. The notion of ontological security, as it is known in the literature, accurately describes the coping mode’s restricted capacity for the creation and protection of well-being. Ontological security leads to a self-limiting form of well-being that has been described as “abnormal normality.” In contrast, psychological safety provides the preconditions for high wellbeing and a safe environment, thus promoting the healthy development of coping and co-creation adequacy.

## Introduction

In this second part of a two-part paper, we apply the separate and complementary ontologies we derived in Part 1 (Andringa & Denham, 2021) to human psychology and efforts to promote well-being. **Coping** and **co-creation** are both manifestations of core cognition. **Core cognition**, we posit, is the foundational cognition shared by all living beings. Part 1 focused on a derivation, mainly from first principles, of core cognition as it pertains to a generic living agent. In this part we transition to human cognition by applying the current conceptualization of core cognition to human identity development, and to two theoretical approaches to enhancing human wellbeing: ontological security (Giddens, 1991; Laing, 1960) and psychological safety (Edmondson, 1999; Clark, 2020). In this process we extend the list of key concepts of core cognition and the two behavioral ontologies with the concepts indicated in **bold.**

In Section 3, we apply the two ontologies of core cognition to identity development and show that the structure of identity development follows logically from the adequate or inadequate use of coping and co-creation. In Section 4, we apply the two ontologies on a metatheoretical level. We demonstrate that the theory of ontological security is a perfect formulation of the coping mode’s ultimately doomed attempt to foster well-being. In contrast, the theory of psychological safety sets up the conditions for human flourishing. We numbered the sections, tables, and figures as to align with Part 1. We have indicated the concepts central to core cognition and these two ontologies in bold and listed their definitions in *Tables 5* and *6.*

## Section 3 — Identity: Learning Co-creation and Coping Adequacy

Previously (Andringa, van den Bosch, & Wijermans, 2015) we have connected the existence of an individual’s unique identity to the self-maintenance of the living state. Here we develop the structure of identity in terms of coping and co-creation adequacy. This leads to an enriched understanding of the interplay between coping and co-creation, and it demonstrates that the conceptual language of core cognition is a productive lens for approaching a well-studied psychological phenomenon. What we describe here connects intimately to the different perspectives on the world that the two brain hemispheres, as described by McGilchrist (2012), produce: *i.e.,* that the left-hemisphere is strongly connected to coping and the right hemisphere to co-creation (Andringa et al., 2015). Editorial constraints prevent us from developing this concept here in detail.

### Identity Development

Berzonsky (1989), quoting Epstein, describes **identity** as a self-generated *theory of me as an actor in the world*, or self-theory: an explanatory structure constructed to explain and plan one’s interactions with the world. It is the basis for understanding one’s position and role in the world and, hence, an expression of one’s **worldview** and **agency.** An adequate self-theory allows one to cope with life’s challenges and respond to opportunities. In return, these enrich one’s self-theory and worldview. One’s self-theory is therefore directly related to how one appraises the world, which links with the way the left and right hemispheres of the brain understand reality (McGilchrist, 2012). Berzonsky (1989) describes this self-theory thus:

a theory that the individual has unwittingly constructed about him- or herself as an experiencing, functional individual … it contains major postulate systems for the nature of the world, for the nature of the self, and their interaction. Like most theories, that self-theory is a conceptual tool for accomplishing a purpose. Major purposes are to optimize the pleasure/pain balance of the individual over the course of a lifetime … and to organize the data of experience in a manner that can be coped with effectively.

Learning to optimize the pain/pleasure balance fits very well with optimizing well-being of the self through self-development of a worldview and an adequate behavioral repertoire for coping and co-creation. According to Berzonsky, the effectiveness of a self-theory can be measured in terms of whether it helps *“to solve the personal problems it was constructed to handle [and …] serve as a framework within which experience and […] relevant information can be meaningfully organized and understood*” (1989). We refer to this (partial) effectiveness as (partial) **adequacy** (see Part 1) and use that to derive the main structure of identity. 

### Identity as Co-creation and Coping (In)adequacy

**Figure 3.**
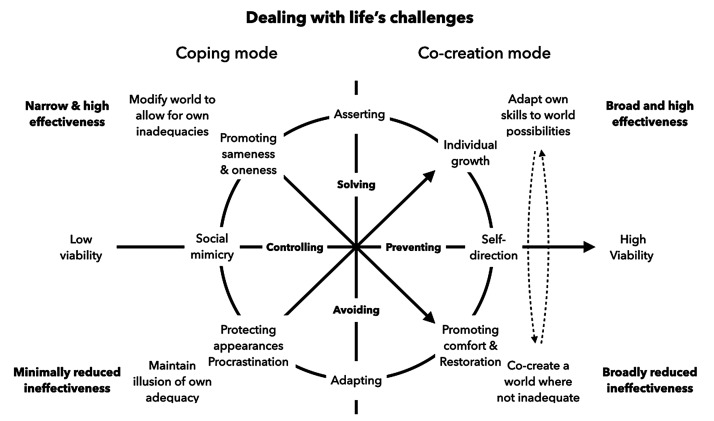
Dealing with life’s challenges. Four attitudes toward problems and challenges (on the main axes), coupled with broad strategies (on the circle), effects on the world, and behavioral (in)effectiveness. The dashed arrows represent life’s key demands: maintaining and increasing viability of self and habitat (Part 1, *Figure 1*). Alternatively attending to both demands implements core cognition.

*Figure 2* in Part 1 described the development of an agent’s behavioral repertoire. In this part we adapt it towards how humans deal with life’s challenges and problems (and indirectly to identity research). In Part 1 we described two main strategies to make the world more predictable and hence more manageable. **Coping** aims to make the world more predictable by reducing its complexity and creating systems (of agents or things) with more predictable behavior, thus bringing threats-to-self under control and promoting security. **Co-creation** makes the world more predictable by promoting unconstrained natural behavior and easy need satisfaction, through promoting and communicating efforts that facilitate and maintain habitat viability and overall safety. We defined a highly adequate agent as one that can *prevent* most problems, and quickly and effectively *solve* what cannot be prevented. Problems (and challenges) that cannot be prevented or solved can be controlled (suppressed) or avoided. These four strategies — preventing, solving, controlling, and avoiding — can be included in *Figure 2* of Part 1 to yield *Figure 3* above.

The main horizontal axis denotes preventing problems (associated with wisdom) as the highest manifestation of self-direction since it leads to high viability of self and habitat (*Figure 1* of Part 1). Its fallback strategy is controlling or reducing (unprevented) problems through social mimicry (Chartrand & van Baaren, 2009) as a manifestation of low self-direction. This is a situation where persistent problems require great effort to handle but are not necessarily successfully controlled and signify low viability. The vertical axis reflects solving problems (associated with intelligence) as a way to assert oneself or, alternatively, avoid them as a way of adapting without changing the situation.

The four quadrants of *Figure 3* correspond directly to those in *Table 3 (*see below*)*, where the combination of attitudes towards problems and challenges defines each of the four table entries that we are going to connect to matching identity statuses (indicated in brackets). In each quadrant we first give a short description in terms of adequacy, and secondly, we describe the associated worldview. 

**Table 3:** Identity as an expression of strategies to deal with life’s challenges. The four cells correspond to the quadrants of Figure 3.

**Controlling & Solving (Identity foreclosure)** Agents modify the world (with great effort) to prevent being confronted with their own inadequacies by promoting a suitable form of sameness and oneness through social mimicry (see Part 1, Coping) which creates an in-group with shared rules (and narratives).	**Preventing & Solving (Achieved identity)** Agents are both adequate problem preventers and problem solvers because they continually self-acquire the skills to benefit most from the possibilities of the world. This allows them to exhibit more or less unconstrained natural behavior.
Their shared worldview enhances in-group effectiveness, but cannot claim realism since it excludes out-group perspectives because it primarily values sameness and oneness.	Their co-creation and coping effectiveness, and hence life-success, prove they have developed and continually maintain a realistic worldview.
**Controlling & Avoiding (Identity diffusion)** Agents have neither co-creation nor coping skills and can only maintain an illusion of agentic adequacy through avoiding challenges or engaging in damage control by behavioral mimicry of (seemingly) successful others.	**Preventing & Avoiding (Identity moratorium)** Agents aim to co-create or select a world where they are not inadequate because it promotes easy need satisfaction and unconstrained natural behavior.
They live in a world of intra- and extra-agentic forces that they neither comprehend nor control, and their worldview is incoherent and inconsistent.	They live in a world that they mostly understand and can handle, but tend to be bothered by long-term problems, which periodically surface, because they lack the skills to address them effectively. In addition, they are blind to the power of complexity reduction and control strategies.

In *Table 3*, the set of behaviors still pertains mainly to general agents, since we limited ourselves to the generalized concepts and formulations derived in Part 1. In the next sections we will introduce, first, the defining two dimensions of the human identity concept, and secondly, we will describe each of the four described identity statuses in relation to what we outlined in *Table 3.*

### The Modern Identity Concept

James Marcia (1967) described late-adolescent development in terms of a transition from “the given” (the dependent) to the (independent) “givers,” and an identity (development) crisis. He described (1966) four identity statuses as combinations of high and low scores on two dimensions: stable commitments and (to use a modern formulation) deliberate self-exploration.

1. Stable commitments indicate that personal strategies are effective and, hence, that one can build — self-directedly — on traces left in the habitat (which is related to concepts like **stigmergy** and **authority**). Since effective strategies are further improved through experience, they do not have to be replaced. This leads to stable, albeit developing, life-strategies and a stable, and effective personality. In *Figure 2* of Part 1, this corresponded to an “upward’’ move towards a more effective behavioral repertoire.
2. Deliberate self-exploration and the development of a self-constructed *theory of me as an actor in the world* is a requirement for the development of a unique self, rather than an identity based on values and beliefs adopted uncritically and unchanged from others (mimicking). The process of deliberate exploration of me-as-an-actor-in-the-world manifests as the broadening of the behavioral repertoire. In *Figure 2* of Part 1 we noted that broadening the behavioral repertoire is more arduous and slower than making it narrowly more effective through mimicking behaviors of those more effective, healthy, or otherwise attractive individuals. But since the broadening contributes to co-creation capacity, it offers higher long-term benefits, and is a preferred choice for individuals who have learned to value co-creation. Valuing these benefits requires the development of co-creation’s basic strategy of discovering, and later using, the unconstrained natural behavior of self, others, and the wider habitat.

The shaping of a unique self occurs on the basis of shared or consensually adopted values, beliefs, and strategies to bootstrap self-development. Actualizing a unique

self requires a shift in one’s perceived locus of causality (PLOC) from external (like social mimicry) to internal: “*The more internalized a value or regulation, the more it is experienced as autonomous or as subjectively located closer to the self*” (Ryan & Connell, 1989, p. 750; Andringa, van den Bosch, & Vlaskamp, 2013). It also manifests self-direction.

PLOC internalization is not so much a rejection of previous values, beliefs, and strategies, but a refinement of these by allowing individual experiences to be enriched and generalized. Hence, they can be applied more flexibly (less rigidly), more context-appropriately (*i.e.*, more realistically), and more proactively with long-term benefits; this is a change from explicit rule-following to the use of experience-based tacit knowledge and self-direction. The combined changes of PLOC from external to internal, from explicit to tacit knowledge use, and from group to individual authority, entail emerging self-direction and liberation from self-limiting constraints, adopted via social mimicry, that warrant characterization as a self-exploration crisis.

Identity research uses past or current self-exploration crises as tell-tale indicators of identity development. In this paper, we connect negotiating or avoiding this crisis to the development (or not) of co-creation adequacy. More precisely, a self-exploration crisis does not indicate co-creation adequacy, but only a co-creation preference; the individual notices its benefit over coping, but is not necessarily adequate yet. Similarly, we connect stable commitments to coping or co-creation adequacy, and the absence of stable commitments to inadequacy. Commitments remain unstable until adequacy is reached. *Table 4* shows this for the four identity statuses we outlined above.

**Table 4:** The four identity statuses

	*No deliberate self-exploration* **Coping preference PLOC external/Low self-direction**	*Deliberate self-exploration* **Co-creation preference PLOC internal/High(er) self-direction**
*Stable commitments*	***Identity foreclosed*** *Self-exploration prevented through adoption of societal norms.*	***Achieved identity*** *Self-exploration crisis negotiated, resulting in well-explored stable identity.*
**Adequate coping**	**Focused on dealing with viability threats to self**	**Effectively improving own and habitatviability**
	The world is unstable and dangerous and needs constant surveillance, control, and forceful efforts to prevent disintegration and becoming totally dysfunctional.	World is full of opportunities and solvable problems and promotes self-development.
	Focus on enforcing complexity reduction of habitat and agent behavioral uniformity through promoting oneness and sameness. An effective, but limited behavioral repertoire.	Focus on opportunities of self and habitat. Self-actualization as an expression of a broad and effective behavioral repertoire.
	They only take responsibility for group-level endorsed actions and procrastinate when forced to self-decide.	They take full responsibility for their actions and tend to address challenges as they come (which benefits development of self and habitat).
	Characteristic insistence on others changing or adapting to protect themselves from exposing their inadequacies: forcing others to mimic them by encouraging or enforcing the adoption of their rules (and narratives).	Corresponds to what Maslow (1954) refers to as self-actualization. It is a state of maximal psychological health and self-development. And it fully implements core cognition.
*No stable commitments*	***Identity diffusion*** *Self-exploration avoided, in combination with a fiuid or unstable self-identity.*	***Identity moratorium*** *Self-exploration crisis (still) in progress, not (yet) leading to a crystalized identity structure.*
**Inadequate coping**	**Contributor to deficient viability of self and habitat**	**Aimed at protecting the conditions for own existence**
	The world is unpredictable and brutal, since actions and outcomes seem unrelated; responsibility for actions is not taken.	The world is sometimes a problematic place but invites continued self-exploration and engagement.
	They focus on strategies that mitigate (public exposure of) inadequacy. Little self-development. Behavioral repertoire is narrow and minimally effective.	They focus on broadening their behavioral repertoire, mastering co-creation strategies and developing a unique identity.
	They take no responsibility for their actions because they can hardly predict the outcomes of their behaviors.	They take responsibility for self-initiated co-creative actions, but procrastinate or evade when faced with serious challenges.
	Their development depends strongly on whether the environment is conducive for it or not. A rich and safe learning environment allows them to progress to the other quadrants, while an unsafe and deprived environment traps them.	Avoidance of challenges deprives them of the learning opportunities to develop high coping skills.

*Note. Words in italics are the deffning properties of the four types of identity statuses (based on Berzonsky, 1989). The identity-status-related core cognition features are in the normal font.*

### Identity from Core Cognition

In the next four subsections we will derive the properties of the four identity statuses described in *Table 4*: achieved, moratorium, foreclosed, and diffusion. Our derivation is based on the framework described in Part 1, and in particular the four-pronged structure to deal with life’s challenges outlined in *Figure 3* and *Table 3.* As has been confirmed (Berzonsky, 1993), we assume no gender differences.

#### Identity Achieved 

An *achieved identity* signifies co-creation and coping adequacy: a rich and effective behavioral repertoire ensures that most problems are avoided, and problems that do occur are dealt with quickly and effectively so that co-creation can resume problem prevention. This involves the individual safely and effectively building on past efforts (**stigmergy**) that produce few unintended and adverse side effects. To the *achieved identity* the world is full of opportunities and solvable problems. And they can and do take responsibility for self-initiated actions. 

Developmentally, the *achieved identity* emerges from a successfully negotiated self-exploration crisis that results in a well-explored stable identity and full self-direction. With the *achieved identity* comes the *informational identity style* that Beaumont and Pratt (2011, p. 174) summarize for achievers as follows:

… they address identity-relevant issues by being skeptical of their self-views, questioning their assumptions and beliefs, and exploring and evaluating information that is relevant to their self-constructions [hence making and keeping their worldview in accordance with the state of the world]. The use of an informational style is positively associated with strategic planning [which includes problem prevention], vigilant decision making, and the use of proactive and problem-focused coping [indicating effective coping and co-creation]. The informational style is also associated with such personal and cognitive attributes as autonomy, openness to experience, introspectiveness, self-reflection, empathy, a high need for cognition, and a high level of cognitive complexity.

These listed properties all facilitate high autonomy, strong self-development, and the effective real-world contributions characteristic of co-creation, as well as high well-being (Berzonsky & Cieciuch, 2016) and wisdom, as we have defined them in Part 1. All in all, this expresses both coping and co-creation adequacy.

#### Identity Moratorium

*Identity moratorium* develops due to a preference for co-creation and coping inadequacy: a (fairly) broad behavioral repertoire ensures that many problems are avoided, but problems which do occur are oft en not dealt with quickly and effectively; the individual cannot (yet) rely on stable and reliable strategies (commit) and instead struggles to develop these. To the person with a *moratorium identity*, the world is a place for continued self-exploration and major problems. He or she experiences an ongoing self-exploration crisis and has a self-development focus that, despite efforts, does not yet lead to a stable identity structure, although it expresses a “limited commitment” (Berzonsky & Cieciuch, 2016) through its co-creation preference.

Although co-creation adequacy might not have been achieved, co-creation is still considered superior to coping and, hence, is the preferred strategy. This means that the person with a *moratorium identity* expresses the strengths of co-creation through a focus on contributing to a high-quality habitat, for which the person can take responsibility. However, the strengths of coping — control of problematic situations and effectively ending problems — are minimally expressed and might, when problem solving is structurally avoided, lead to toxic situations. This leads to less time for co-creating than the *achieved identity* status, and comfort, defined as an absence of apparent pressing problems, is highly valued.

People with a *moratorium identity* express many of the features of the informational identity style, but to a lesser degree due to their lower coping skills, which also leads to lower well-being than the *achieved identity* style (Berzonsky & Cieciuch, 2016).

#### Identity Foreclosure

*Identity foreclosure* is the identity status that is central for the next section, so we elaborate it in this subsection. Identity foreclosure combines co-creation inadequacy with adequate coping. Co-creation inadequacy leads to structurally unprevented problems, but coping adequacy ensures that these are managed with effort — *i.e.,* controlled — so that they do not (usually) spin out of control. The concept of **security**, defined as threats brought and kept under control, describes this. The associated worldview is one of an unstable and dangerous world that needs constant surveillance, control, and the need for forceful efforts to prevent disintegration and becoming totally dysfunctional. This motivates the individual with a *foreclosed identity* more oft en than not (although limited meta-cognition ensures that they are unaware of this).

*Identity foreclosure* corresponds to prevented (foreclosed) self-exploration through the uncritical adoption of consensual norms (Berzonsky, 1989; Marcia, 1966) and social mimicry. The dominance of the coping mode leads to favoring in-group level rules and, in general, shared (explicit) knowledge over individual (implicit) knowledge. Foreclosed individuals aim to adopt and express shared rules and narratives with great diligence, and they actively promote the adoption of their shared worldview. Neither the body of shared rules nor the single shared worldview is explored since it is adopted on the basis of superficial effectiveness and social mimicry rather than deliberation on its effectiveness and context appropriateness. The associated worldview is therefore often at odds with actual states of reality, thus perpetuating the body of unprevented problems that have to be controlled.

The resulting strict adherence to the norm and an insistence of oneness and sameness — generating an ingroup — effectively curtails agent and habitat diversity. This is considered moral and responsible behavior because it is intended to manage the threats that keep the coping mode activated. Ironically, “foreclosed” individuals see little value in co-creation’s preventative strategies and in questioning its associated assumptions and beliefs. Instead, they view them as **out-groups**: individuals who violate sameness and oneness, and hence, frustrate coordinated coping. This means that the “foreclosed” individual is blind to (superior) strategies that might structurally prevent the problems they try so hard to keep from spinning out of control. Hence, more often than not, the threats and problems persist, which locks this identity status into a self-perpetuated **coping trap.**

Groups of foreclosed individuals manifest a **social level coping trap** that, through their insistence on coordinating the behaviors of others, threatens to dominate the habitat. Groups of foreclosed individuals have the only identity status that insists on others changing and conforming. Their (unspoken) motto is: “We are right and you have to adapt your behavior to match ours.” They feel righteous because they have no access to perspectives and worldviews other than their own, and they lack the tools to judge the merits of out-group insights. Hence, they see only potential harm in out-group strategies.

Worse, they are particularly insensitive to arguments more nuanced or personal than rule-following and other forms of social mimicry. In fact, they prefer cognitive closure (Webster & Kruglanski, 1994) in answering questions on a given topic, over continued uncertainty, confusion, and ambiguity. An even more profound formulation of their motto is: *“Out-group diversity, such as nuanced thoughts and self-directed behaviors, activates a sense of inadequacy in me, through raising doubt on my shared belief system. Diversity, therefore, must be suppressed.”*

Individuals with a *foreclosed identity* express a particular form of information processing known as the normative identity style. We referred to this in an autonomy development context as cognition for control, order, and certainty (Andringa et al., 2015). The normative identity style is a form of information processing that latches onto the familiar, the standardized, the expected, and whatever has direct utility (McGilchrist, 2012). As such, it prefers representations that have been stripped of ambiguities and have been made fixed, uniform, invariant, and static. And in its problem-solving, it denies inconsistencies and instead latches on to a single, normabiding, in-group-promoting solution, and an associated narrative that has been coupled with totalitarianism and authoritarianism (Beaumont, 2008; Berzonsky, 1989). The normative identity style of the foreclosed identity has been summarized as follows:

Normative individuals more automatically internalize and conform to the standards and expectations of significant others. Discrepancies between information about how they are and their normative standards evoke feelings of guilt and concern about avoiding failure [to be a good in-group member]. Their primary aim is to defend and maintain existing self-views [to protect a shared worldview that promotes coordinated action]. (Berzonsky, 2008, pp 646)

Normative individuals report high levels of identity commitment as well as dispositional characteristics such as agreeableness, conscientiousness [both facilitating rule following], and extraversion [promoting the adoption of the shared rules]. However, they also report low levels of openness and introspectiveness [which forecloses further identity development], Normative individuals have been found to employ avoidant coping strategies, to procrastinate in the face of [individual] decisions, to have a high need for structure and a low tolerance for ambiguity, and to be conservative, authoritarian, and racist in their sociocultural views (Beaumont, 2009, p. 97)

Karen Stenner (2005) summarizes the foreclosed identity’s characteristic urge to reduce complexity as “Intolerance to diversity = Authoritarianism x normative fear level,” where authoritarianism is a measure of identity foreclosure. She describes **normative threats** as threats to oneness (shared authority) and sameness (shared values and rules). In particular, she lists questioned or questionable authorities and values, disrespect for leaders or leaders unworthy of respect, and lack of conformity with or consensus in group norms and beliefs (Stenner, 2009, p. 143): all correspond to a disintegration of oneness and sameness. This summarizes the existential threat felt by those with a foreclosed identity when their only strategy to secure well-being — behavioral diversity reduction through (imposed) limits on agency — is frustrated. But when they do not feel threatened, people with a foreclosed identity manifest intermediate levels of well-being (Berzonsky & Cieciuch, 2016), since they are generally able to maintain problems and threats at manageable levels. All in all, this identity status expresses high coping adequacy and co-creation inadequacy.

#### Identity Diffusion

The fourth identity status is referred to as *identity diffusion* and is characterized by inadequate co-creation and inadequate coping. People with this status live in a world of unprevented and unsolvable problems, with dynamics that they do not comprehend, with rules they do not know how to apply skillfully, and where effort and hoped-for outcomes are only weakly related. Given their low adequacy, their well-being depends predominantly on environmental factors. For people with identity diffusion the world is unpredictable and often brutal despite the best of intentions. Hence, they procrastinate in the face of self-decision and will not take responsibility for their actions.

*Identity diffusion* is characterized by prevented or avoided self-exploration in combination with a fluid or unstable self-identity. While aiming to improve their well-being, people with *identity diffusion* are often confronted with the consequences of their own inadequacy. Their intentions are good; their realization is not. And one often ends up in, or even self-perpetuates, low viability states. And without the benefit of self-exploration, they do not understand the causes of their problems. Much more than with the other identity statuses, people with identity diffusion live in a random (and brutal and unjust) world of problems in which they cannot take responsibility for their actions. This contrasts with achievers who live in a world of opportunities to be explored and responsibly realized. Beaumont and Pratt (2011, p. 174) describe the associated identity style thus:

A diffuse-avoidant identity style is associated with procrastination and attempts to evade identity conflicts and decisional situations as long as possible [all due to self-perceived inadequacy and mitigating efforts to prevent adverse outcomes and being exposed as inadequate]. …The use of a diffuse-avoidant style is characterized by low agreeableness, conscientiousness, introspectiveness, [complicating rule following] and cognitive complexity [indicating a shallow worldview], and high neuroticism. A diffuse-avoidant style is also associated with less adaptive cognitive and behavioral strategies, such as using avoidant coping strategies, engaging in task-irrelevant behaviors, expecting to fail, having a low feeling of mastery, and performing less strategic planning. [all indicating coping and co-creation inadequacy]

This description clearly demonstrates that people with a diffusion identity exhibit a narrow range of marginally effective or ineffective behavioral options that lock them into this status and curtail their well-being (Berzonsky & Cieciuch, 2016). They express both coping and co-creation inadequacy. Nevertheless, self-development occurs, and they can, although later than others, adopt narrowly effective strategies (towards the *foreclosed identity* status), develop self-exploration abilities (towards the *identity moratorium* status), or both (towards the *achieved identity* status).

### Psychology from Core Cognition

In Section 3, we have connected the four combinations of co-creation, coping, adequacy, and inadequacy to the four identity statuses. The psychological literature has derived the properties of these statuses and the associated information-processing styles via careful experimentation and observation (in particular the copious body of research by Berzonsky). But to our knowledge, we are the first to derive the structural properties of identity from first principles (in fact, this might be a first for any phenomenon in psychology). This provides evidence that human psychology is indeed rooted in the core cognition shared by all life.

We also suggest a phylogenetic scaffolding which has coping and co-creation (as essentials of core cognition) as the foundation; identity status and associated information-processing styles building on this; and then personality traits like the Big Five on top. This is not new; two personality meta-traits, referred to as plasticity and stability (DeYoung, Peterson, & Higgins, 2002), have been proposed with a similar scaffolding model. More recently, DeYoung (2015) posited the underlying role of plasticity and stability in a cybernetic Big Five theory of *goal-directed* adaptive systems. This is similar to DeYoung’s proposal, although its goal-directedness suggests that it pertains predominantly to the coping mode.

## Section 4 — Two Routes to General Well-being

This section addresses two routes to social well-being. There are many routes to prospective well-being; in fact, all self-help literature and political, economic, or religious ideologies propose them. We have selected the “ontological security” framework and a recent formulation of “psychological safety” to represent very clear, actionable, and precisely-worded coping and co-creation alternative approaches to general well-being. In this section we will apply our core cognition framework as a metatheoretical lens to inspect the “theories” described below. This requires us to focus on the mindsets that spawned the theories behind either the coping or co-creation ontology.

Ontological security and psychological safety refer to seemingly similar, but essentially different, concepts of avoiding danger. We address that first.

### Safety versus Security

**Safety** is a situation or state with positive indicators of the *absence* of viability threats. It is a precondition for co-creation and for achieving and maintaining the higher levels of well-being. Adequately co-creating agents self-organize a shared habitat while minimizing tension and conflict and maximizing natural unconstrained behavior. Their co-creation adequacy prevents danger, harm, or injury because it allows agents to focus on restoration and growth.

In humans, the role of this absence of threats is exemplified by the difference between calm and boring sonic environments, *i.e.,* the presence or absence of audible safety (Andringa & Lanser, 2013; Van den Bosch et al., 2013; van den Bosch et al., 2018). Similarly, squirrels infer safety from bird chatter (Lilly, Lucore, & Tarvin, 2019). In addition, recent studies on how to improve the well-being of people with dementia (where reduced higher cognition opens a window to more basic processing) show marked reduction in problematic behavior by just reducing the prevalence of (unpleasant) sounds which are indicative of unsafety (Koster et al., submitted 2021).

While safety is a precondition for co-creation, security is the objective of coping. The Concise Oxford dictionary defines **security** as *“the state of being free from danger or threat.”* Here we sharpen this definition to “a state where viability-threats-to-self have been brought under control,” to stress its deliberate manifestation in coping. In our modern societies, increased coping prevalence is manifested by the changing role of the (national) security state from a sole focus on international war, to include policing domestic and foreign populations (Andreas, 2001; Raskin, 1976). It is no longer other states that are the problem, but our own domestic population represents a security threat to be controlled (Zedner, 2003). Similarly, organizations can both trust or distrust worker autonomy. Distrust of worker autonomy promotes coercive formalization of work as bureaucracy (Adler & Borys, 1996; Andringa, 2015). These examples suggest that “greater security” does not necessarily signify “more safety.” It just indicates more coordinated behaviors and stricter control of potential (even imagined) threats.

“Security” and “safety” play central roles in the two attitudes towards the creation of well-being that we will discuss. Security provides actively maintained **short-term** sanctuary by controlling threats to viability and through enhancing control over diversity and complexity to promote oneness and sameness. It is a manifestation of coping, associated with the *foreclosed identity* status, and the normative identity style. In contrast, safety provides and creates environmental conditions conducive to **long-term** well-being through avoiding problems, actively signaling the absence of threats, and maintaining an environment for restoration, growth, and, in general, co-creation. It is associated with the *achieved identity* status and its informational identity style.

### Ontological Security

#### The Origins of Ontological Security

Creating “security” is associated with reducing fear by excluding “the unknown” and controlling whatever activates feelings of inadequacy. An **in-group**, as a defining feature, shares common adequacy limits and aims to control the environment to make it more orderly, stable, structured, predictable, and therefore, less threatening to the in-group by imposing limits on agency via routines, norms, and rules. This method of creating well-being is defined by theorists in Sociology and International Relations as “ontological security,” and we interpret it here as a perfectly formulated attempt by individuals in the coping mode to improve their well-being. However, since it has the coping mode’s limitations, it can only improve low well-being to a situation of no symptoms. It cannot bring about the higher levels of well-being achievable by co-creation.

The concept of **Ontological Security** was popularized by Anthony Giddens who described it as the secure feeling an individual derives from attaining *“on the level of the unconscious and practical consciousness, ‘answers’ to fundamental existential questions* [*i.e.*, problems] *which all human life in some way addresses”* (1991, p. 47). However, the origins of ontological security can be found in Laing’s *The Divided Self* (1960). For Laing, psychoanalysis is about helping the patient reconstruct his identity, or “way of being himself in his world,” so as to show no overt symptoms (Laing, 2010, p. 25). Laing states that individuals who have a “partial or almost complete absence” of this “person-in-the-world” theory are more likely to develop psychosis and schizophrenia (1960, p. 39). He describes such individuals thus:

His identity and autonomy are always in question. He may lack the experience of his own temporal continuity. He may not possess an overriding sense of personal consistency or cohesiveness. He may feel more insubstantial than substantial, and unable to assume that the stuffhe is made of is genuine, good, valuable (Laing, 1960, p. 42).

Here Laing describes the diffusive identity status; inadequate, unskilled, with an underdeveloped self-theory, and with an inadequate behavior repertoire that is often ineffective, and potentially or progressively disconnected from the inner (self) and outer reality. He does not describe the other identity statuses because, as a mental health practitioner, his concern is with removing the *symptoms* of schizophrenia and psychosis. In the absence of a diagnosis, he has no tools to promote optimal mental health, or maximize human potential or self-actualization (Maslow, 1954). Hence, the concept of ontological security emerges exclusively from the logic of the coping mode, as its formulation and conceptualization are ignorant of co-creation.

Giddens (1991) provides a sociological interpretation of Laing’s insights, arguing that our identity and autonomy, and by extension our ontological security, depend on our ability to trust in social narratives and routines in which we are contextually embedded, and through which our identity is constituted. Adhering to norms and routines means individuals are not *“obsessively preoccupied with their contingent, and fragile nature”* (Rossdale, 2017, p. 371).

For Giddens the aim of gaining security is not to “accept” reality, or broaden and deepen adequacy by developing a richer self-theory through exploration of self and the world. Instead, its purpose is *“to create ontological reference points”* which simplify reality so that inadequate agents can deal with *“the contexts of day-to-day life”* (1991, p. 48) without learning and growth towards full self-actualization. In the terms laid out in *Figure 2* in Part 1, the aim is to make the behavioral repertoire more effective through social mimicry via the adoption of behaviors of (authoritative) others. It does not promote broadening the scope of behaviors. Hence it promotes both the normative identity style as much as it promotes authoritarianism.

#### Normative Threats to Ontological Security

According to Giddens, norms and routines which coordinate behavior, provide us a *“cognitive and emotional anchor”* from which (inadequate) individuals derive the “trust” (Giddens, 1991, p. 36) that continuity and stability will prevail in everyday relations, so that they are not confronted with their own inadequacy. Routines rely heavily on a complex body of shared knowledge, constituting a societal status quo that can be mimicked wholesale: taken-for-granted local practices, cultural narratives, institutional structures, and “common” knowledge (Berger & Luckmann, 1991, p. 35). In other words, the status quo is the “*anchor”* from which security is derived. Ontological security is associated with (normative) individuals who have acquired a narrow but conditionally effective skill set for coping; they lack the behavioral breadth to deal with a world that is not under control of their in-group, and they feel an existential threat when so confronted.

This is a direct reference to Stenner’s book *The Authoritarian Dynamic* (2005) that we addressed in Section 3 on the *foreclosed identity* status, which predicts authoritarianism. Individuals with a foreclosed identity status respond with intolerance to diversity when confronted with normative threats, and hence they promote common authority (oneness) and shared values (sameness). The most threatening conditions to oneness and sameness “are questioned or questionable authorities and values, for example, disrespect for leaders or leaders unworthy of respect, and lack of conformity with or consensus in group norms and beliefs” (Stenner, 2009, p. 143).

Here, the normative “threat” to oneness and sameness concerns the condition of the self, more than of the perceived disturbance: “the self is unsure what to expect of the new: the exact boundary and inclusion or exclusion of the newcomer are not clear” (Chernobrov, 2016, p. 586). In general, the unfamiliar “new” exposes the individual’s inadequacy; uncertainties regarding the unfamiliar “hamper calculation and increase risk, jeopardize perceived or actual security, or signal indeterminacy and lack of meaning” (Chernobrov, 2016, p. 582). Consequently, security is concerned with maintaining one’s relationship with the environment as it is, via purging it of sources of uncertainty. This makes sense since inadequate agents are not equipped with the skills to understand or deal with the unknown outside of in-group-controlled environments. (See Part 1, Section 2 “Coping,” and the subsection on the foreclosed identity style).

#### Attempting Well-being via Ontological Security

The process of gaining ontological security is the process of becoming partially adequate via adoption of normative strategies (the mimicking of status quo behaviors) to minimize viability threats. As Mitzen (2006, p. 342) puts it, *“for theorists of ontological security, individual identity is formed and sustained through relationships”* with significant others, as is expected of people with the foreclosed identity status and the associated normative identity style (Berzonsky, 2008), who express the coping mode structurally and preferentially.

Berger and Luckmann (1991, p. 71) refer to the adoption of empowering routines and norms as “habitualization.” **Habitualization** is the consolidation of routines via reference to socially constructed symbols, myths, and heritage — shared knowledge — that sustain an in-group identity, which, in the words of Kinvall, provides *“a guide for future actions”* (2004, p. 756). Norms, rules, and routines impose in-group level limits on agency and reduce diversity (Rossdale, 2017), while increasing the probability of intended outcomes. All moves to achieve or retain ontological security enact limitations which restrain political critique and possibility, and securitizes subjectivity (Rossdale, 2017, p. 370). Interestingly, habitualization activates resistance in the form of the psychological phenomenon of **reactance**: the motivation to liberate oneself from limits on self-directed behaviors (Miron & Brehm, 2006).

Individuals don’t only ascribe meaning to their own normative experience but are able to “*unite* [...] *in a way that promotes order and predictability”* (Gergen, 2001, p. 18 in Skey, 2010). The resulting less complex environment no longer confronts inadequate individuals with their inadequacy because it is, for them, more manageable and predictable, and hence it appears and is appraised as less threatening. However, this complexity reduction also stipulates that security is achieved via adherence to the status quo at the expense of personal freedom, options for self-directed contributions (by implementing authoritarianism), and diminished congruence with the actual state of reality. Inevitably, the façade of a less complex environment needs continual and effortful maintenance so as not to crumble in the face of reality. The weaker the façade appears, the stronger the normative threat, and the more frantically the façade is defended and diversity suppressed.

Any out-group identity is constructed via **othering, *“****which denotes exclusionary and antagonistic differences”* (Rossdale, 2017, p. 374; Kinvall, 2004). Inadequate co-creators are only familiar with and comfortable in their own in-group context, so they construct the unfamiliar individual’s identity comparatively to their own, with a focus on difference rather than similarity (Skey, 2010), and exclude everything, even things of great value, when they fall outside the knowledge base of the in-group. This results in the construction of identities and routines as *“relative to other identity constructions’’* (Kinvall, 2004, p. 762), making each in-group seemingly incompatible and inherently separate. By viewing each other as stereotyped members of a group relegated to a foreign status (Skey, 2010), they create **out-groups.** As the foreign is threatening to inadequate co-creators, out-groups perceived as “different” are almost always seen as a problem (threat-to-self). This process of othering leads to polarization, which traps the in-group deeper in the coping ontology.

The resulting security is short term because it relies on exerting continual control through the suppression of unwanted diversity (which exposes one’s inadequacies). *“The process of achieving (or seeking to achieve) ontological security frequently involves forms of exclusion and othering which may be both violent and counterproductive”* (Rossdale, 2016, p. 370). As there is only coping, there is zero-gain; *“Increasing ontological security for one person or group* [...] *is thus likely to decrease security for those not included”* (Kinvall, 2004, p. 763). Routines and rules are advantageous to the in-group, as they stipulate order and increased predictability. However, members of the out-group are disadvantaged by these rules and, in turn, are threatened and feel insecure. In-groups provide the out-group with grievances: exclusion, suppression, supervision, et cetera. Silke (2008, p. 112) asserts that if *“marginalized groups are discriminated against or* [...] *believe that there is discrimination, then there will always be sections within such communities who will be receptive to radical ideologies,”* thereby jeopardizing the security of the environment. *“Empirically and normatively* [ontological security] *push*[es] *us in the wrong direction”* (Nesbitt-Larking, 2016, p. 13).

Searching for security by relying on in-group norms and routine also can distract from real-world threats, and actually make the group less safe and effective. When speaking of the failure of commercial organizations such as Radio Shack, Blockbuster, or Kodak, Clark (2020) stated: “*These organizations were filled with large numbers of highly intelligent people, and yet they all fell prey to competitive threats that were hiding in plain sight. The countervailing strategies their competitors put in place were not mysterious. They were, in fact, obvious. What these organizations failed to do was challenge the status quo and disrupt themselves.* [...] *They allowed the status quo to fossilize and would not allow themselves to change it.”* In other words, protecting the status quo might actually degrade one’s situation in a changing world. This is the fate of individuals existing exclusively under the coping mode’s limitations: it may postpone death, but it provides no guarantee for being or becoming well.

#### Pathological Normality as the Coping Mode’s Ideal of Well-Being

What does the ideal of ontological security look like? It would be a symptomless perfect adaptation to a carefully controlled environment, protected from everything that might freak out the foreclosed personality. Aldous Huxley (1958), quoting Erich Fromm, noted that symptoms means conflict, which indicates

that the forces of life which strive for integration and happiness are still fighting. The really hopeless victims of mental illness are to be found among those who appear to be most normal. “Many of them are normal because they are so well adjusted to our mode of existence, because their human voice has been silenced so early in their lives, that they do not even struggle or suffer or develop symptoms as the neurotic does.” They are normal not in what may be called the absolute sense of the word; they are normal only in relation to a profoundly abnormal society. Their perfect adjustment to that abnormal society is a measure of their mental sickness.

These millions of abnormally normal people, living without fuss in a society to which, if they were fully human beings, they ought not to be adjusted, still cherish “the illusion of individuality,” but in fact they have been to a great extent de-individualized. Their conformity is developing into something like uniformity. But “uniformity and freedom are incompatible. Uniformity and mental health are incompatible too … Man is not made to be an automaton, and if he becomes one, the basis for mental health is destroyed.”

This corresponds with Maslow’s observations about the suppression of an essential human core, of which he says “even when its existence is denied, it never goes away, even in a sick person; and is constantly trying to get out. Discipline, deprivation, frustration, pain, and tragedy are necessary because these experiences foster and fulfill his inner nature” (Maslow, 1968, pp. 3-4). Maslow argued that “psychologically speaking, that which designates a normal human being is in reality a psychopathology of the average. It depicts a lifestyle that is so widespread and nondramatic that we don’t even notice it ordinarily. In general, this normal life is one of general phoniness, illusion, and fear; showing that it is a sickness that is widely spread.” (1968, p. 16).

Striving for ontological security then fosters a psychopathology of the average: a state of marginal well-being and psychological emptiness which is the best that coping can produce: it is **pathological normality.** Unfortunately, it is also what Hannah Arendt (1963) refers to as the “banality of evil” in her description of the normality of Eichmann who “would have had a bad conscience only if he had not done what he had been ordered to do.” 

The question then is how to promote and achieve higher levels of well-being.

### Psychological Safety

**Psychological safety** is a term that was first derived in teamwork research where it helped to predict which teams would work well and which would not. Psychological safety promotes interpersonal risk taking (Edmondson, 1999) and signifies a change from a defensive and self-protective team member to being a fully collaborating member without any motivation to self-protect.

Feeling safe is conditioned on positive **indicators of safety.** Safety is an outcome of successful previous behaviors (both coping and co-creation), and signifies that all is well. Therefore, safety signifies high adequacy, pervasive optimization (wisdom), inclusion, and **wu wei.** In such an environment, changes are attended to before they become pressing problems because in an inherently safe environment enough individuals have adequate skills to approach and adapt to the (natural dynamic of the) unfamiliar, without feeling threatened and defensive.

Whereas ontological security has a focus on maximizing environmental mastery through minimizing habitat complexity, **psychological safety** has a focus on maximizing agentic contributions in ways that benefit the whole. Via anthropological fieldwork conducted on organizations *“from every sector of society,”* Clark (2020) described the concept this way: Psychological safety is a condition in which you feel

1) included; 2) safe to learn; 3) safe to contribute; and 4) safe to challenge the status quo — all without fear of being embarrassed, marginalized, or punished in some way. Step four exemplifies interpersonal risk-taking most clearly.

Clark (2020) argued that the progression toward psychological safety is derived from the natural sequence of human needs; the pre-conditions required for co-creation to occur. Maslow’s hierarchy of needs (1943) has five stages (physiological, security, belongingness, esteem, and self-actualization) that correspond directly to Clark’s progression towards psychological safety. The most basic needs for Maslow are physiological: food, water, and shelter; these needs are not included in Clark’s psychological safety because each one “is a postmaterialist need” (2020). Maslow’s next three stages (security, belongingness, and esteem) are the needs that Clark conceptualized as the three needs on which psychological safety depends. Psychological safety “is no less a human need than food or shelter,” since it is the manifestation of the need for agentic self-preservation, which has as much to do with “social and emotional needs as physical ones” (Clark, 2020). Once the basic needs of food, water, and shelter are met, psychological safety becomes a priority so that an individual’s maximum potential is unleashed; self-actualization and co-creation preconditions are satisfied.

#### Fours Steps of Psychological Safety

The first step for psychological safety is inclusion. The concept of inclusion underpins the difference between safety and security. When creating well-being via security, in-group membership is always conditional. In-group members feel unthreatened because of sameness and oneness: security derived by the suppression of diversity. When creating well-being via safety, membership in the community is *“based on the sole qualiffcation that they possess fiesh and blood”* (Clark, 2020) (which is easily generalized to include all living agents).

For *inclusion safety*, agents must be equipped with the skills to negotiate the unfamiliar by extending both respect and permission. By respect, Clark means the average level of esteem agents afford to each other; how much agents value and appreciate the unfamiliar. By permission, Clark means the degree to which the group allows the unfamiliar to influence them; that all, including newcomers, are permitted to participate as members of the community. Permission and respect are important affordances that agents grant one another in order to create an environment that provides safe passage for maximizing agentic potential and cultivating confidence, resilience, and independence (Clark, 2020). All in all, unconditional membership allows full access to the co-creation side of *Figures 2* and *3.*

The next level of safety is *learner safety.* Learner safety implies that you feel safe to participate, engage with the discovery process, ask questions, and make mistakes. The transition to learner safety means the agent faces the anxiety of the unknown (all signs of in-groupiness) and is not limited by it.

As individuals feel increasingly safe in a nurturing environment that offers respect and permission, we enter the stage of *contributor safety.* This is the stage where the individual is invited to participate as a full-fledged member of the community, and his/her esteem needs are fulfilled (Maslow, 1943). The agent’s contributions are successful; he feels adequate, skilled, and valuable. Hence, he gains self-esteem and, in turn, is increasingly respected by the community. Contributor safety emerges when the individual has acquired skills and is able to apply them adequately to produce shared benefits. The community has to provide both encouragement and appropriate autonomy to the agent (Clark, 2020). If the individual is hampered by discrimination, prevailing norms, internal bias, a lack of empathy, or general aloofness, he or she is denied contributor safety.

The final and crucial stage of psychological safety is *challenger safety*; an individual feels free to challenge the status quo without fear of retribution or reprisal (Clark, 2020). Challenger safety enables individuals to overcome the pressure to conform and, hence, can enlist themselves in co-creative processes; improvement, innovation, development, and hence communal growth (the more-than-zero-sum feature of co-creation).

While allowing and promoting challenger safety is a defining feature of psychological safety, challenges to the status quo are exactly what is to be suppressed from an (ontological) security perspective. Here the in-group’s focus is on protecting and defending the rules, routines, and norms that define the ingroup by suppressing diversity. Since ingroups feel inadequate under normative threats (challenges to sameness and oneness), any challenge is interpreted as an assault on precisely what constitutes the normative and authoritarian identity. And that is why suppressing diversity is incompatible with psychological safety.

Psychological safety is achieved via maximizing member contributions so that

1) members are equipped with the skills to confidently negotiate the unknown and unfamiliar; and 2) new and current members feel free to join, learn, contribute, and criticize freely, and, therefore, never harbor the motivation to threaten the well-being of the **community.** The result is a community or habitat presenting a high concentration of safety indicators in the form of unscripted contributions to the community. The progression towards psychological safety and fulfilling the natural sequence of human needs provides a recipe for co-creative well-being and growth.

### Metatheoretical Considerations

The very formulation of the theory of ontological security shows that it is possible to formulate, with the best of intentions, a framework that is almost guaranteed to lead to a deeply pathological state of individual and societal non-development. Ideally this results in a situation of no symptoms, populated by individuals perfectly adapted to a world that is kept within the limits of their underdeveloped co-creation adequacy: *pathological normality.* Additionally, maintaining a world within tight constraints is arduous and wasteful compared with societal developing of the skills to deal with full real-world complexity, threats, and opportunities, as effective co-creation allows to be done.

Although this argument might be convincing for some, it is not acceptable for in-groups (*i.e.,* authoritarians), especially not for those under normative threats, who simply assume that out-groups must either comply with their in-group rules or be dealt with otherwise (eliminated, removed, or made irrelevant). Due to the absence of self-exploration and the associated lack of broadening of the behavioral repertoire towards co-creation adequacy, this means that the coping worldview is simply not rich enough to adequately assess its own limitations, let alone understand full human potential. Possibly, this also characterizes the formulators of ontological security, since they seem unaware of the existence of co-creation. The formulation of psychological safety, on the other hand, expresses co-creation very clearly, but it is concurrently aware of coping and its limitations because it straddles co-creation and coping skills.

**Figure 4.**
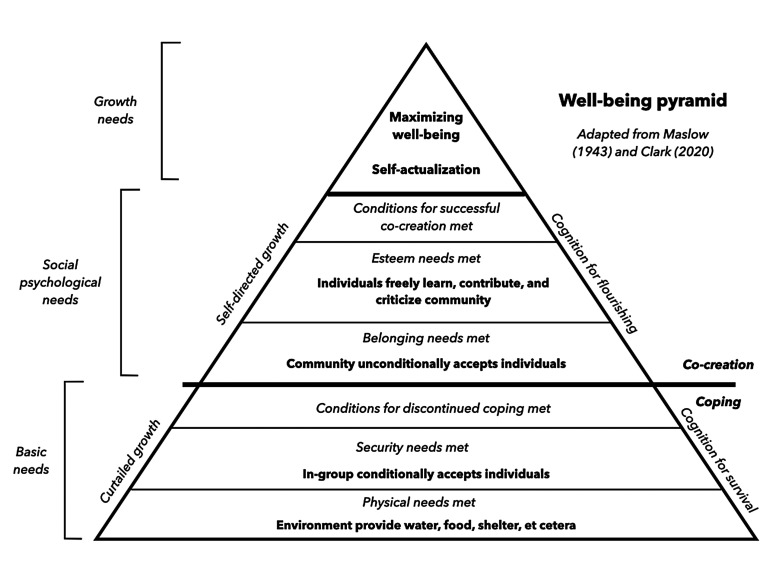
Well-being pyramid. The key transition is from conditional acceptance by ingroups to unconditional acceptance by a (diverse) community.

Clark’s (2020) description of the preconditions for psychological safety, in combination with the internal logic of the ontological security framework, inspired us to produce a metatheoretical summary (*Figure 4*) which builds on Maslow’s pyramid of needs (1943). The key transition in this well-being pyramid occurs between the lower level and access to self-directed growth towards self-actualization (Maslow, 1954).

The transition occurs when coping strategies stop being dominant and co-creation takes over, while coping remains a valuable fallback to address pressing problems quickly and effectively. This corresponds to a change from in-groups that conditionally accept individuals — namely if and only if they accept the in-group worldview and, hence, direct and curtail their behaviors according to shared adequacy limits — to a community that offers unconditional **acceptance** to individuals and allows them to learn, contribute, and criticize. This makes the group diverse in its ability to solve problems and realize opportunities, and it offers ample context for individual and community growth. The transition also corresponds to a switch from “cognition for control, order, and certainty” (coping) to “cognition for exploration, disorder, and possibility” (co-creation) that we described in *Figure 2* of Andringa et al. (2013b).

## Conclusion

In this (long) two-part paper we aimed to derive central aspects of cognition from first principles and called the resulting framework core cognition. We summarized the key terms we used in this part in *Table 5.* We derived two separate forms of cognition: 1) coping, which addresses pressing problems and hence is aimed at their termination; and 2) co-creation, which is aimed at optimizing everything in the context of everything else and aimed at its perpetuation. We assert that both strategies are essential; but it is the interplay of their strengths that, somewhat unexpectedly but logically, leads to the dominance of one of them: co-creation. Because we derive our conclusions from studying generic living agents, we claim that our results not only pertain to human well-being, but to well-being in general: well-being for all living beings, and by extension, for the biosphere.

**Table 5:** Core cognition key terms used in this part

	Core cognition key concepts with definition used in this paper
Core Cognition	The cognition shared by all life
Agent	“An autonomous organization that adaptively regulates its coupling with its environment and contributes to sustaining itself as a consequence.” (Barandiaran, Di Paolo, & Rohde, 2009, p. 1)
Behavior	Agent-initiated and context-appropriate activities with expected future utility that counteract life’s precariousness and maximizes agent and habitat viability.
Viability	Probabilistic distance from death (*i.e.*, discontinued agency)
Agency	The ability to self-maintain viability (through need satisfaction) for survival and thriving
Cognition	The ability to select behavior in the service of the agent’s continued existence and flourishing.
Coping and co-creation	Two complementary forms of cognition. Coping is in the service of continued existence and flourishing in the service of flourishing. (These two forms of cognition are opposed in *Table 2*)
Stigmergy	Building on the constructive traces that past behaviors left in the environment (increasing habitat viability)
Authority	Expressing stigmergy
Habitat	The environment from which agents can derive all they need to survive (and thrive) and to which they contribute to ensure long-term viability (of self and others), Note that we use the term habitat to include other agents, but to exclude the agent. Hence, we can speak of agent + habitat to refer to the whole of existence relevant to the agent
Habitat viability	A measure of the degree to which the habitat can satisfy the conditions for agentic existence *(i.e.*, satisfies its needs)
Well-being	Process of co-creation leading to high viability agents, increased habitat viability, and long-term protection of the conditions on which existence depends. Note that this is a process, not a state or the evaluation of a state.
Context	Agent’s assessment of the (current) state of the habitat
Behavioral repertoire	The set of context-appropriate behaviors the agent has access to
Learning	The process to extend the behavioral repertoire and tune its effectivity to the context
Worldview	The set of all that an agent takes as reliable (true) enough to base behavior on.
Appraisal	A worldview-based motivational response to the perceived viability consequences of the present.
Realism	A measure of whether individual behavior leads to intended and/or viability enhancing outcomes
Identity	A theory of me-as-actor-in-the-world

The different purpose and character of coping and co-creation lead to two complementary ontologies of cognition (*Table 6*), each of which follows its own internal logic and has separate key concepts. Coping expresses cognition for survival, and co-creation expresses cognition for flourishing. The differences between the goals and internal logic of coping and co-creation means that individuals who approach the world from these different logics do not understand each other at all. Coping and co-creation adequacy has to be learned from real-world interactions on top of innate abilities (to acquire these). But not everyone becomes adequate in both.

**Table 6:** Ontologies of survival and thriving (expanded from Part 1)

Ontology of survival (coping)		Ontology of thriving (co-creation)	
Languishing	Low viability state as the outcome of a pattern of ineffective or limited behaviors	High viability state as the outcome of a pattern of broadly effective behaviors	Flourishing
Danger	Agent appraisal of viability threats, entailing a *reduction* of the set of context appropriate behavioral options to include only those that allow the agent to survive.	Agent appraisal of the *absence* of viability threats, entailing an *enlarging* of the set of context appropriate behavioral options to include more options that allow the agent to thrive	Safety [freedom]
Problem	A perceived threat to agent viability that activates a pressing need and hence motivates reactive behavior	A perceived possibility to improve (agent or habitat) viability which hence motivates proactive behavior	Opportunity
Coping	The reactive fallback mode of behavior aimed at protecting agent viability by ending problem states. Quick and effective deactivation of coping is the measure of success of the coping mode	The pro-active default mode of behavior aimed at producing indirect viability benefits through habitat contributions that improve the conditions for future agentic existence	Co-creation
Reactive behavior	Behavior in response to perceived threats to viability	Behavior aimed at setting up or protecting the conditions for co-creation	Proactive behavior
Main mode of cognition: Intelligence	The ability to solve problems (or end states of pressing needs)	The ability to avoid problems and co-create: (Also: The balancing skills to contribute to the biosphere)	Main mode of cognition: Practical wisdom
Coping trap (Coping failure)	The continual or predominant activation of the coping mode of behavior through ineffective or counterproductive problem-solving strategies.	Prolonged or near continual activation of co-creation	Successful co-creation
Inadequacy	The tendency to self-create, prolong, or worsen problems that keep on activating the coping mode. An inadequate agent is predominantly coping, but unsuccessful in ending the activators of coping.	The skill to avoid problems or end them quickly so that coping is rare and co-creation prevalent. An adequate agent is predominantly a co-creator	Adequacy
Coping adequacy	The skill to solve pressing problems (ending the need to cope) or mitigate their impact through control of the environment and constraining agency (continuing coping)	The skill to avoid and end prob lems through harmonizing relations and (inter-agent) conflict mitigation	Co-creation adequacy
Power	The ability to realize intended outcomes by effortfully shaping and controlling the habitat and the activities of the agents that comprise it. Exercising power is a way to be authoritative.	Effortless action aimed at being authoritative through harmonizing a diversity of agentic interests by promoting natural agentic dynamics and development	Wu wei
Security	A situation or state where viability threats-to-self are brought under control	A situation or state with positive indicators of the absence of viability threats	Safety
Well-being-short term	Self-evaluation of one’s agentic viability	Holistic self-valuation of one’s own and the habitat’s viability	Well-being-long term
Ontological security	The secure feeling an individual derives from attaining “on the level of the unconscious and practical consciousness, ‘answers’ to fundamental existential [problems] which all human life in some way addresses” (Giddens, 1991)	Self-realizing one’s full individual potential	Self-actualization
Rules of ontological security	I am accepted when I contribute to sameness and oneness I learn rules and routines of my in-group I adhere to in-group roles I protect the in-group against unmanageable diversity	I can join freely I can learn freely I can contribute freely I can criticize freely	Rules of psychological safety
Habitualization	The consolidation of routines via reference to socially constructed rules and routines, sustaining a group identity and the security on derives from in-group membership.	The motivation to liberate oneself from imposed limits on self-guided behavior and the restoration of the safety associated with co-creative processes	Reactance
In-group	A group of individuals sharing similar limits on adequacy (and worldview)	A group of individuals that each freely and self-guided contribute whatever benefit	Community
Out-group	Individuals who are not in-group and hence frustrate coordinated coping	their adequacy can bring	
Othering	The process of assigning individuals with other or less limits to adequacy to out-groups (possibly disgust — driven)	Unconditional acceptance	Acceptance
Pathological normality	Complete and symptomless adaptation to a world shaped through coping that imposes limits on individual agency and self-development	The ability to co-create and cope in the service of full self-development	Healthy normality
Normative threat	Threats to oneness (shared authority) and sameness (shared values and rules)	Perceivable indications of other agents engaged in unforced activities	Indicators of safety

Section 3 showed that the four combinations of coping, co-creation, adequacy, and inadequacy underlie the structure of identity in humans, and shed new light on why the various identity statuses have their characteristic properties and how this connects to how each status approaches information. In particular, the combination of adequate coping and inadequate co-creation leads to individuals who strive to control their environment by promoting a single shared world-view and a single set of appropriate behaviors; this is to prevent it spinning out of their control, and hence exposing their narrow basis of adequacy. This is the authoritarian mindset as reflected by the foreclosed identity and its normative information processing style. Stenner’s concept of the authoritarian dynamic (2005) — intolerance of diversity equals the degree of authoritarianism times the normative threat level — follows directly from these properties.

In Section 4, we applied core cognition as a metatheoretic tool. We concluded that striving to realize what is known in the literature as “ontological security” is a precise expression of the coping mode’s (limited and doomed) capacity for well-being. In fact, we concluded that ontological security leads to a self-limiting form of well-being — pathological normality — that has been described as “*abnormal normality”* by Huxley (1958) and Fromm, and as *“the pathology of the average”* by Maslow (1968, p. 16). By contrast, Maslow’s understanding of well-being and self-actualization exemplifies co-creation. We concluded that psychological safety provides the preconditions that maximize well-being and the **healthy normality** of developing coping and co-creation adequacy.

Already in 1973, Newell wondered about psychology’s ability to produce wonderful scientific papers (Newell, 1973). He asked himself the question of whether psychology would have achieved *“a science of man”* by his assumed retirement age in 1992, or whether another multi-decade period of paper production would be necessary to *“home in on the essential structure of the mind.”* Newell concluded: *“I am worried that our efforts, even the excellent ones I see occurring here, will not add up”* (to the formulation of *“a science of man”*). He speculated: *“Maybe we are reaching the day of the theorist in psychology, much as it exists in other sciences such as physics. Then the task of putting things together falls to them and experimentalists can proceed their own way”* (Newell, 1973, p. 306)

Perhaps we have contributed a unifying perspective — by assuming core cognition shared by all life — that helps make sense of the huge body of data that psychology has compiled. We hope we have, and we will investigate this further by applying core cognition insights to such diverse domains as happiness and education research; separate brain systems such as dual type processing (Evans & Stanovich, 2013) in the left & right hemisphere (McGilchrist, 2012); the structure of values (Fontaine et al., 2008); and radicalization and extremism. Our hope is not to fragment knowledge and understanding any further, but to find more ways in which to unify the acquired body of evidence into a more manageable framework.
